# Single Nucleotide Polymorphisms in *PLCE1* for Cancer Risk of Different Types: A Meta-Analysis

**DOI:** 10.3389/fonc.2018.00613

**Published:** 2018-12-11

**Authors:** Xiaoying Li, Xuelian Li, Min Jiang, Wen Tian, Baosen Zhou

**Affiliations:** ^1^Department of Epidemiology, School of Public Health, China Medical University, Shenyang, China; ^2^Key Laboratory of Cancer Etiology and Prevention, Liaoning Provincial Department of Education, China Medical University, Liaoning, China

**Keywords:** *PLCE1*, cancer, polymorphism, susceptibility, meta-analysis

## Abstract

**Background:** Recent studies have investigated the relationships between *PLCE1* polymorphisms and cancer susceptibility. However, some findings lack consistency.

**Objectives:** In the current study, we conducted a meta-analysis to more accurately evaluate the relationships between *PLCE1* (rs2274223, rs3765524, rs753724, rs11187842, and rs7922612) single nucleotide polymorphisms (SNPs) and risk for different types of cancer.

**Methods:** We performed a comprehensive search strategy in PubMed, Web of Science, Medline, EMbase, and Scopus for articles available until 19 March 2018. A total of 54 case-control studies comprising 17,955 cases and 20,400 controls were included in the current meta-analysis, which together comprised a total of 32 publications. The pooled odds ratios (ORs) with 95% confidence intervals (CIs) were used to evaluate relationships between the *PLCE1* polymorphisms and cancer susceptibility. All statistical analyses were performed using Stata 11 software.

**Results:** Results of the meta-analysis demonstrated that the rs2274223 polymorphism showed a significant correlation with increased overall cancer susceptibility (AG vs. AA: OR 1.168, 95% CI 1.084–1.259; GG vs. AA: OR 1.351, 95% CI 1.163–1.570; AG+GG vs. AA: OR 1.193, 95% CI 1.103–1.290; GG vs. AA+AG: OR 1.262, 95% CI 1.102–1.446; G vs. A: OR 1.163, 95% CI 1.089–1.242). Results of subgroup analysis showed that the rs2274223 polymorphism was associated with higher risk for esophageal cancer and gastric cancer relative to colorectal cancer and head and neck cancer. In addition, the rs2274223 polymorphism was found to be associated with increased cancer risk, especially among the subgroups comprising Asians, studies with population-based controls, studies employing the TaqMan genotyping method, and studies consistent with Hardy-Weinberg equilibrium (HWE). The association between the rs3765524 polymorphism and reduced overall cancer risk was detected in one specific genetic model (CT vs. CC: OR 0.681, 95% CI 0.523–0.886). Results of subgroup analysis showed that the rs3765524 polymorphism was associated with cancer risk in a specific genetic model among the subgroups of colorectal cancer, esophageal cancer, Asians, studies with population-based controls, and studies consistent with HWE. However, relationships among the *PLCE1* rs753724, rs11187842, and rs7922612 polymorphisms and tumor risk were not identified.

**Conclusions:** Results of the current meta-analysis suggested that *PLCE1* (rs2274223, rs3765524) polymorphisms are associated with cancer susceptibility.

## Introduction

Cancer has become a major threat to public health worldwide (1). In 2018, there is a predicted 1,735,350 new cancer cases, which are equivalent to over 4,700 new cancer diagnoses each day in the United States, which correspond to an expected 609,640 cancer deaths (2). In addition, cancer has become the leading cause of death in China (3). Therefore, there is an urgent need to investigate cancer, identify relevant biomarkers, and develop strategies for active prevention and early diagnosis and treatment. Cancer is well-established to be the result of a combination of genetic and environmental factors. In the last few decades, extensive experimental and epidemiological findings demonstrated the close association between genetic alterations and tumor risk (4). Single nucleotide polymorphisms (SNPs), the most common form of gene alteration in the human genome, refers to single-nucleotide variations with distribution frequencies that are >1% in the population.

Phospholipase C epsilon1 (*PLCE1*), which is located on chromosome 10q23, is a member of the phospholipase C protein family (5). In 2010, the results of genome-wide association studies indicated that *PLCE1* is associated with cancer risk (6, 7). Since then, multiple researchers investigated the relationship between *PLCE1* polymorphisms and cancer risk. Cui et al. (8) explored the association between *PLCE1* polymorphisms and risk for esophageal squamous cell carcinoma. Li (9), Zhang (10) and other authors investigated the relationship between *PLCE1* polymorphisms and colorectal cancer risk. Yuan (11), Malik (12) and other authors investigated the association between *PLCE1* polymorphisms and gastric carcinoma risk. Sharma (13) and other authors showed that *PLCE1* polymorphisms were associated with susceptibility to developing gall bladder cancer. Among all studies that investigated *PLCE1* polymorphisms and cancer susceptibility, the SNPs rs2274223, rs3765524, rs753724, rs11187842, and rs7922612 were five of the most extensively studied polymorphic loci. However, we noted significant differences in the results, sample size, race, or selection of controls among the different studies. In addition, the latest meta-analysis on the relationship between the rs2274223 polymorphism and the overall cancer risk was published in 2015 (14). Furthermore, to the best of our knowledge, no studies conducted meta-analysis of the association of rs3765524, rs753724, rs11187842, and rs7922612 polymorphisms with overall cancer risk. Therefore, in the present study, we summarized all currently qualified case-control studies to obtain a more accurate understanding of the relationship between the *PLCE1* polymorphism rs2274223 and overall cancer risk [(15) studies were added to the current meta-analysis from the meta-analysis published in 2015 (14)]. And we firstly performed a meta-analysis of the association between the rs3765524, rs753724, rs11187842, and rs7922612 polymorphisms and cancer risk in the overall population.

## Methods

### Literature Search

We carried a comprehensive search strategy to retrieve qualified publications from PubMed and Web of Science until 19 March 2018. The search queries comprised a combination of the Medical Subject Headings (MeSH) and the following keywords: (rs2274223 OR rs3765524 OR rs753724 OR rs11187842 OR rs7922612) OR (*PLCE1* OR PLCE OR PPLC OR NPHS3) and (cancer OR tumor OR carcinoma OR neoplasm OR malignancy). In addition, we searched literatures from Medline, EMbase, and Scopus, as complementary data. The references of qualified articles or other reviews were additionally searched. For publications with no available original data, we contacted the authors to ensure that data from all qualified literatures were included in the current meta-analysis. The authors of three out of six publications responded.

### Inclusion Criteria and Exclusion Criteria

The inclusion criteria for qualified literatures were as follows: (a) The studies evaluated the associations between *PLCE1* polymorphisms (rs2274223 or rs3765524 or rs753724 or rs11187842 or rs7922612) and cancer risk. (b) The study had available genotyping data required for the calculation of the odds ratios (ORs) with 95% confidence intervals (95% CIs). (c) The studies were case-control studies. (d) Studies were complete original articles. Exclusion criteria of qualified literatures were as follows: (a) Articles did not estimate the relationships between the *PLCE1* (rs2274223, rs3765524, rs753724, rs11187842, or rs7922612) polymorphisms and cancer susceptibility. (b) The article was a repeated publication. (d) Primary data were missing and were not obtained after contacting the authors. (e) The subjects were not human. Two researchers independently retrieved the literature. In the case of different views in the selected literature, the two researchers discussed to reach an agreement or the decision was made by an independent researcher (Xuelian Li).

### Reporting Items

Two investigators independently gathered data from each selected article, including the first author, year, country, ethnicity, tumor type, genotyping methods, the source of control, number of cases and controls, and the *P*-values of the HWE test of the controls. In the case of different views, the two researchers reached an agreement through discussion.

### Quality Score Assessment

All qualified literatures were individually assessed by two researchers based on the Newcastle-Ottawa scale (NOS) (16). The assessment results indicated that all selected literatures were of relatively high quality (all NOS scores were ≥6). In addition, the two researchers assessed the quality of the studies using the STREGA (strengthening report of genetic association studies) quality score system (15). All STREGA scores were >12, which indicated that the quality of the studies was moderate-high or high.

### Statistical Analysis

Hardy-Weinberg equilibrium (HWE) was examined by performing a Chi-square test in the controls. Heterogeneity was evaluated by conducting *Q*-test and *I*^2^-test. In addition, the pooled ORs with 95% CIs were calculated based on the random effects model when heterogeneity was significant (*I*^2^ > 50%) (17). Otherwise, pooled ORs with 95% CIs were calculated according to the fixed-effects model (18). The pooled ORs with 95% CIs were used to evaluate relationships between the *PLCE1* polymorphisms (rs2274223, rs3765524, rs753724, rs11187842, and rs7922612) and cancer susceptibility. To investigate the potential sources of heterogeneity across different studies, stratification and meta-regression analyses were conducted. Sensitivity analyses were carried out to evaluate the stability of the results. The effect of publication bias was evaluated using Begg's funnel plot (19) and Egger's test (20). All the above analyses were performed using Stata 11 software. *P* < 0.05 was considered statistically significant.

## Results

### Study Characteristics

**Figure 1:**
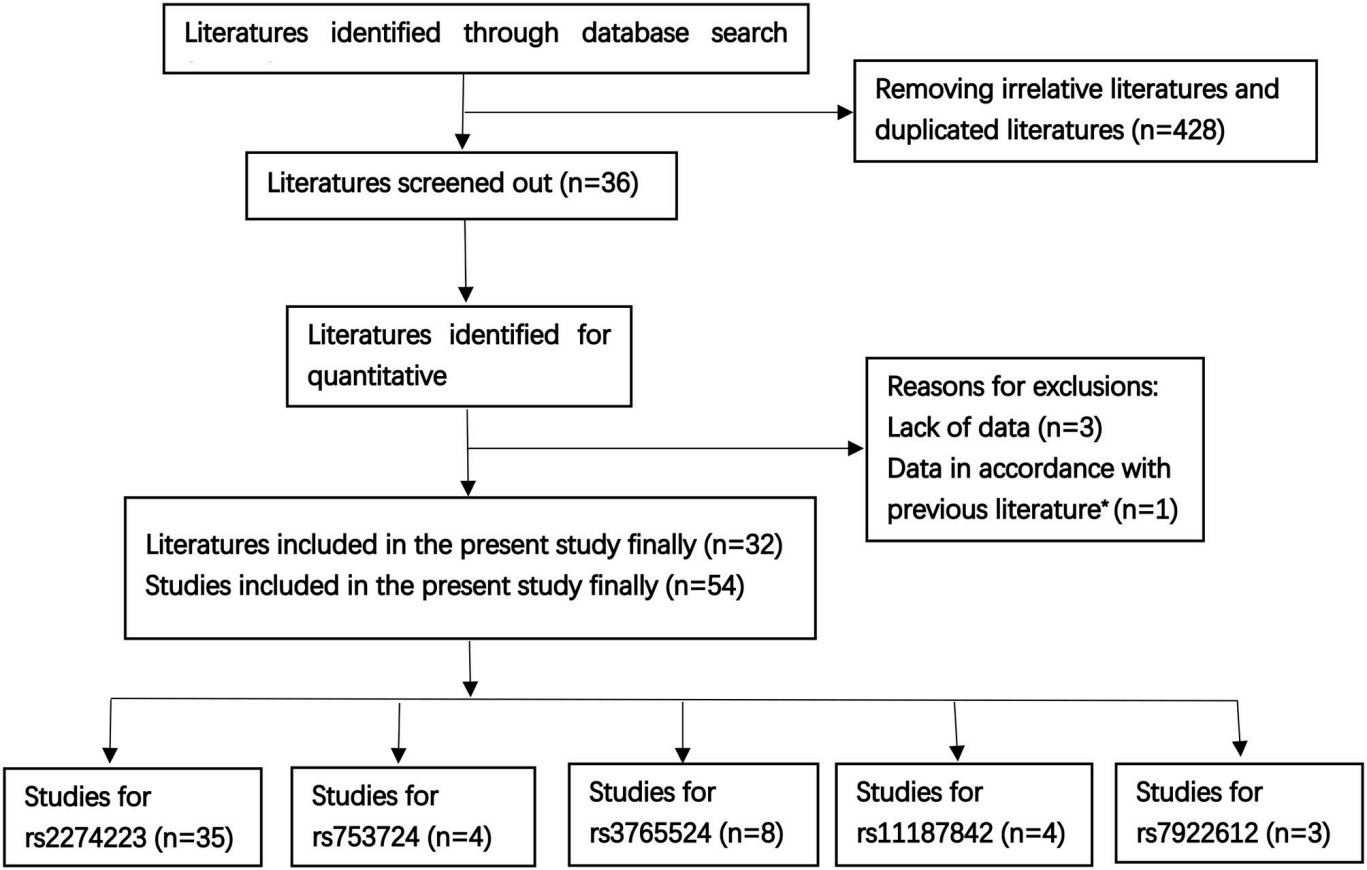
The workflow of the enrollment [Literatures sources: PubMed *n* = 112, Web of Science *n* = 201, other literatures were supplemented by Medline, EMbase, and Scopus, as well as the references of qualified literatures or other reviews; previous literature^*^ (13)].

A total of 32 literatures were eventually included based on the above described comprehensive search strategy. The workflow of the enrollment in the meta-analysis is presented in Figure 1. A total of 54 case-control studies comprising 17,955 cases and 20,400 controls were included in the 32 publications. Five target SNPs were investigated in the current meta-analysis. The main characteristics of the 54 case-control studies and the genotype distribution information of the five polymorphisms are summarized in Table 1. The rs2274223, rs3765524, rs753724, and rs11187842, rs7922612 polymorphisms were involved in 35 (8, 9, 12, 21–45), eight (8, 9, 12, 34, 38, 45–47), four (8–10, 48), four (8–48), and three studies (12, 31, 38), respectively. The different cancer types investigated included gastric cancer, head and neck cancer, colorectal cancer, esophageal cancer, gall bladder cancer, and lung cancer. Of all case-control studies, only genotype frequencies of three studies among the controls were not consistent with HWE (45, 46). A total of 36 studies involved Asians; 16 studies involved Caucasians; one study involved Africans; and one study involved individuals of mixed ancestry. Meanwhile, 35 studies were hospital-based, and 17 studies were population-based. All studies were case-control studies.

**Table 1:** Characteristics of studies.

**First author**	**Year**	**Country**	**Ethnicity**	**Cancer type**	**Source of control**	**Genotyping methods**	**Cases (*****n*****)**			**Controls(*****n*****)**			**HWE (*P*)**
							**AA**	**AB**	**BB**	**AA**	**AB**	**BB**
**rs2274223**													
Zhang et al. (21)	2011	China	Asian	GC	PB	TaqMan	867	664	134	1122	643	83	>0.05
Ma et al. (22)	2011	USA	Caucasian	HNC	HB	TaqMan	477	506	114	504	474	111	>0.05
Li et al. (9)	2012	China	Asian	CRC	HB	MassArray	155	71	5	180	92	20	>0.05
Zhou et al. (23)	2012	China	Asian	EC	PB	PCR	248	227	42	291	191	280	>0.05
Gu et al. (24)	2012	China	Asian	EC	HB	MassArray	202	147	30	233	119	19	>0.05
Hu et al. (25)	2012	China	Asian	EC	HB	TaqMan	594	400	67	754	399	58	>0.05
Bye et al. (26)	2012	South African	African	EC	Mixed	TaqMan	140	208	70	302	411	137	>0.05
Bye et al. (26)	2012	South African	Mixed	EC	HB	TaqMan	78	130	46	310	408	139	>0.05
Palmer et al. (27)	2012	Poland	Caucasian	GC	PB	TaqMan	107	138	44	154	166	56	>0.05
Palmer et al. (27)	2012	USA	Caucasian	GC	PB	TaqMan	132	150	24	86	107	17	>0.05
Palmer et al. (27)	2012	USA	Caucasian	EC	PB	TaqMan	30	18	4	86	107	17	>0.05
Palmer et al. (27)	2012	USA	Caucasian	EC	PB	TaqMan	44	50	13	86	107	17	>0.05
Wang et al. (28)	2012	China	Asian	GC	PB	TaqMan	600	399	60	791	390	59	>0.05
Cui et al. (8)	2013	China	Asian	EC	HB	MassArray	108	93	21	193	121	12	>0.05
Yuan et al. (29)	2013	China	Asian	HNC	PB	TaqMan	301	170	30	547	300	32	>0.05
Duan et al. (30)	2013	China	Asian	EC	PB	PCR	193	150	38	281	123	16	>0.05
Sharma et al. (31)	2013	North Indian	Caucasian	GBC	HB	PCR	174	229	13	111	98	16	>0.05
Dura et al. (32)	2013	Netherlands	Caucasian	EC	PB	PCR	42	38	6	279	247	54	>0.05
Dura et al. (32)	2013	Netherlands	Caucasian	EC	PB	PCR	118	116	24	279	247	54	>0.05
Li et al. (33)	2013	China	Asian	GC	HB	TaqMan	197	122	16	217	109	8	>0.05
Chen et al. (34)	2013	China	Asian	EC	PB	MALDI-TOF MS	97	84	19	178	111	11	>0.05
Yang et al. (35)	2014	China	Asian	EC	HB	TaqMan	172	122	19	209	96	9	>0.05
Malik et al. (12)	2014	Kashmir	Asian	GC	HB	PCR	54	45	9	100	78	17	>0.05
Piao et al. (36)	2014	Korea	Asian	EC	PB	PCR	153	140	29	909	684	107	>0.05
Kupcinskas et al. (37)	2014	Lithuania and Latvia	Caucasian	GC	HB	PCR	94	126	30	91	116	34	>0.05
Umar et al. (38)	2014	India	Caucasian	EC	HB	PCR	162	120	11	168	127	19	>0.05
Wang et al. (39)	2014	China	Asian	CRC	HB	TaqMan	228	161	28	269	128	19	>0.05
Song et al. (40)	2014	Korea	Asian	GC	PB	HRM	1818	1197	230	909	684	107	>0.05
Kupcinskas et al. (41)	2015	Lithuania and Latvia	Caucasian	CRC	HB	TaqMan	77	91	24	147	173	56	>0.05
Jia et al. (42)	2015	China	Asian	EC	HB	MassArray	194	140	24	190	104	11	>0.05
Sun et al. (43)	2015	China	Asian	GC	PB	PCR	405	254	33	514	226	34	>0.05
Dong et al. (44)	2015	China	Asian	LC	HB	iMLDR and direct sequencing	106	46	7	106	73	7	>0.05
Dong et al. (44)	2015	China	Asian	GC	HB	iMLDR and direct sequencing	93	56	18	106	73	7	>0.05
Dong et al. (44)	2015	China	Asian	EC	HB	iMLDR and direct sequencing	65	39	5	106	73	7	>0.05
Ezgi et al. (45)	2016	Turkey	Caucasian	CRC	HB	PCR	142	48	10	176	54	0	<0.05
**Rs3765524**													
Li et al. (9 ]	2012	China	Asian	CRC	HB	MassArray	156	70	5	180	92	20	>0.05
Chen et al. (34)	2013	China	Asian	EC	PB	MALDI-TOF MS	108	78	14	176	108	16	>0.05
Cui et al. (8)	2013	China	Asian	EC	HB	MassArray	120	87	15	191	118	17	>0.05
Umar et al. (38)	2014	India	Caucasian	EC	HB	PCR	167	113	13	177	125	12	>0.05
Malik et al. (12)	2014	Kashmir	Asian	GC	HB	PCR	58	42	8	109	74	12	>0.05
Mou et al. (46)	2015	China	Asian	GC	NA	Universal tagged arrays	104	64	23	82	29	17	<0.05
Ezgi et al. (45)	2016	Turkey	Caucasian	CRC	HB	PCR	78	112	10	84	108	18	<0.05
Qu et al. (47)	2017	China	Asian	EC	PB	PCR	362	169	19	385	150	15	>0.05
**rs753724**													
Li et al. (9 ]	2012	China	Asian	CRC	HB	MassArray	169	57	5	203	76	13	>0.05
Yuan et al. (48)	2012	China	Asian	GC	HB	MassArray	196	80	3	225	63	8	>0.05
Cui et al. (8)	2013	China	Asian	EC	HB	MassArray	133	85	4	246	77	3	>0.05
Zhang et al. (10)	2015	China	Asian	CRC	HB	MassArray	194	66	16	296	79	9	>0.05
**rs11187842**													
Yuan et al. (48 ]	2012	China	Asian	GC	HB	MassArray	196	80	3	225	63	8	>0.05
Li et al. (9)	2012	China	Asian	CRC	HB	MassArray	169	57	5	203	76	13	>0.05
Cui et al. (8)	2013	China	Asian	EC	HB	MassArray	151	68	3	253	71	2	>0.05
Zhang et al. (10)	2015	China	Asian	CRC	HB	MassArray	174	42	14	279	76	8	>0.05
**rs7922612**													
Sharma et al. (31 ]	2013	North Indian	Caucasian	GBC	HB	PCR	67	234	115	24	122	79	>0.05
Malik et al. (12)	2014	Kashmir	Asian	GC	HB	PCR	47	47	14	90	85	20	>0.05
Umar et al. (38)	2014	India	Caucasian	EC	HB	PCR	133	132	28	134	153	27	>0.05

*GC, Gastric cancer; HNC, Head and neck cancer; CRC, Colorectal cancer; EC, Esophageal cancer; GBC, Gallbladder cancer; LC, Lung cancer; HB, Hospital-based; PB, Population-based; NA, Not available; AA AB BB: AA AG GG for rs2274223, CC CT TT for rs3765524, GG GT TT for rs753724, CC CT TT for rs11187842, CC CT TT for rs7922612*.

### Meta-Analysis of the Relationship Between the *PLCE1* rs2274223 Polymorphism and Cancer Risk

**Figure 2:**
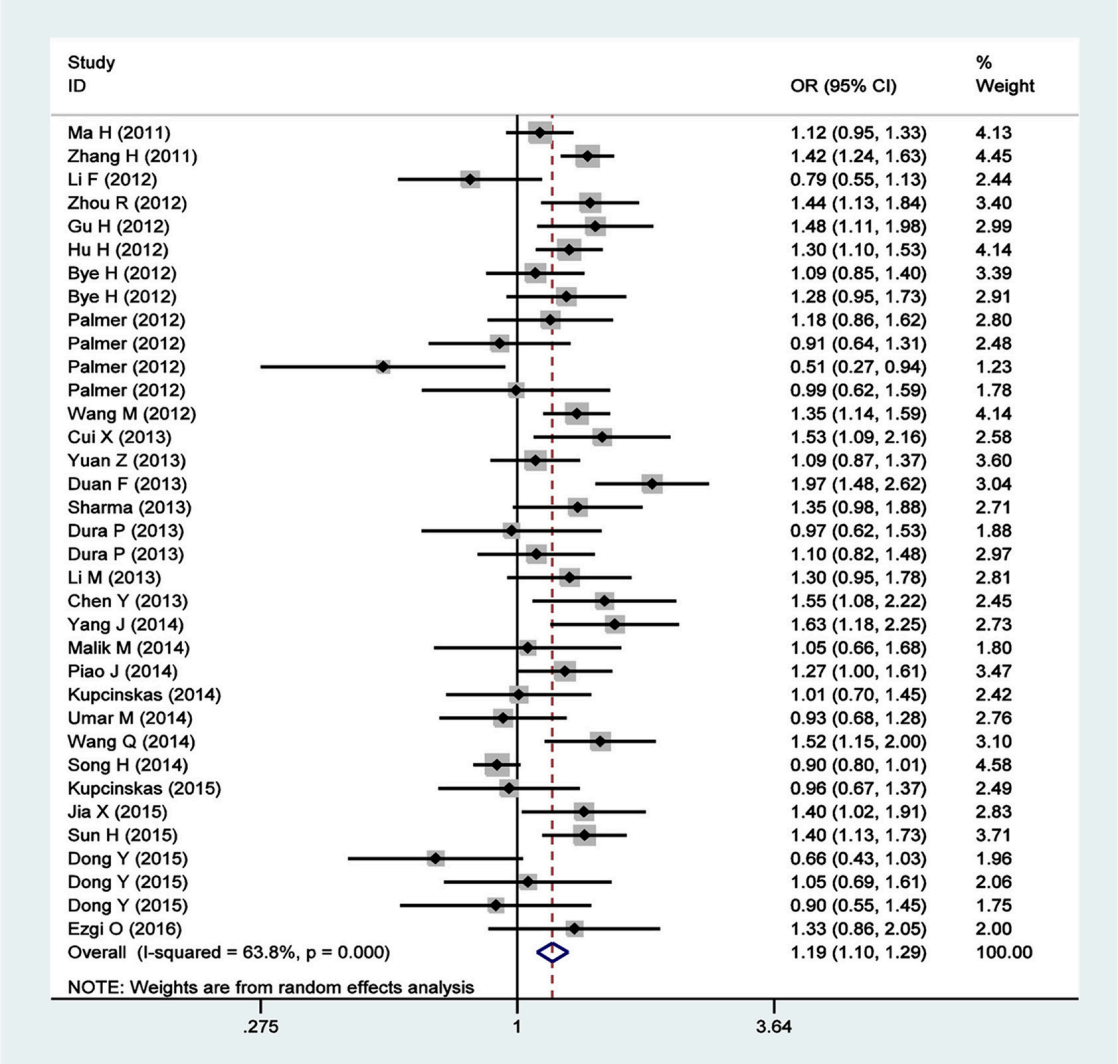
Forest plots for rs2274223 polymorphism and cancer risk in dominant model (AG+GG vs. AA).

A total of 35 qualified case-control studies were included in this meta-analysis, which assessed the relationship between the *PLCE1* rs2274223 polymorphism and cancer susceptibility. We evaluated heterogeneity and selected the random effects model or the fixed-effects model based on the results of *Q*-test and *I*^2^-values. Results of the meta-analysis of the relationship between the *PLCE1* rs2274223 polymorphism and cancer risk are shown in Table 2 and Figure 2. Results showed a correlation between the rs2274223 polymorphism with significantly increased overall cancer susceptibility in all genetic models [AG vs. AA: OR 1.168, 95% CI 1.084–1.259 (*P* < 0.001); GG vs. AA: OR 1.351, 95% CI 1.163–1.570 (*P* < 0.001); AG+GG vs. AA: OR 1.193, 95% CI 1.103–1.290 (*P* < 0.001); GG vs. AA+AG: OR 1.262, 95% CI 1.102–1.446 (*P* = 0.001); G vs. A: OR 1.163, 95% CI 1.089–1.242 (*P* < 0.001)]. To further study the association between rs2274223 polymorphism and cancer risk, we carried out stratified analyses according to cancer type, ethnicity, the source of control, genotyping methods, and HWE. The results of subgroup analyses are also shown in Table 2. Results according to the cancer type indicated that the rs2274223 polymorphism was associated with a higher risk of gastric cancer in four genetic models [GG vs. AA: OR 1.317, 95% CI 1.041–1.667 (*P* = 0.022); AG+GG vs. AA: OR 1.163, 95% CI 1.002–1.350 (*P* = 0.047); GG vs. AA+AG: OR 1.271, 95% CI 1.114–1.449 (*P* < 0.001); G vs. A: OR 1.144, 95% CI 1.018–1.286 (*P* = 0.023)]. Meanwhile, the rs2274223 polymorphism was related to a significantly increased risk of esophageal cancer in all genetic models [AG vs. AA: OR 1.247, 95% CI 1.157–1.344 (*P* < 0.001); GG vs. AA: OR 1.542, 95% CI 1.247–1.907 (*P* < 0.001); AG+GG vs. AA: OR 1.266, 95% CI 1.133–1.415 (*P* < 0.001); GG vs. AA+AG: OR 1.356, 95% CI 1.192–1.544 (*P* < 0.001); G vs. A: OR 1.226, 95% CI 1.112–1.351 (*P* < 0.001)]. However, we found no statistically significant associations between the rs2274223 polymorphism and risks of head and neck cancer and colorectal cancer. The results of subgroup analyses according to ethnicity indicated that the rs2274223 polymorphism increased cancer susceptibility in Asians [AG vs. AA: OR 1.221, 95% CI 1.102–1.352 (*P* < 0.001); GG vs. AA: OR 1.665, 95% CI 1.381–2.006 (*P* < 0.001); AG+GG vs. AA: OR 1.270, 95% CI 1.142–1.412 (*P* < 0.001); GG vs. AA+AG: OR 1.465, 95% CI 1.316–1.632 (*P* < 0.001); G vs. A: OR 1.251, 95% CI 1.145–1.366 (*P* < 0.001)]. However, the association between rs2274223 polymorphism and cancer risk was not identified in Caucasians. The results of subgroup analyses based on the source of control showed that the rs2274223 polymorphism was associated with significantly increased risk of tumor in hospital-based subgroup [AG vs. AA: OR 1.188, 95% CI 1.106–1.277 (*P* < 0.001); GG vs. AA: OR 1.289, 95% CI 1.009–1.647 (*P* = 0.042); AG+GG vs. AA: OR 1.206, 95% CI 1.126–1.291 (*P* < 0.001); G vs. A: OR 1.150, 95% CI 1.050–1.259 (*P* = 0.003)]. Meanwhile, the statistically significant relationship between the rs2274223 polymorphism and cancer susceptibility was also detected in the population-based subgroup [AG vs. AA: OR 1.169, 95% CI 1.028–1.329 (*P* = 0.017); GG vs. AA: OR 1.448, 95% CI 1.184–1.770 (*P* < 0.001); AG+GG vs. AA: OR 1.203, 95% CI 1.054–1.373 (*P* = 0.006); GG vs. AA+AG: OR 1.352, 95% CI 1.205–1.516 (*P* < 0.001); G vs. A: OR 1.184, 95% CI 1.066–1.316 (*P* = 0.002)]. The results of subgroup analyses based on genotyping methods indicated that rs2274223 polymorphism might increase tumor risk in all genetic models in TaqMan subgroup. In addition, the rs2274223 polymorphism was related to a significantly higher risk of tumor in three genetic models in the PCR subgroup [AG vs. AA: OR 1.278, 95% CI 1.163–1.405 (*P* < 0.001); AG+GG vs. AA: OR 1.290, 95% CI 1.178–1.412 (*P* < 0.001); G vs. A: OR 1.183, 95% CI 1.041–1.344 (*P* = 0.010)]. However, the rs2274223 polymorphism was associated with tumor risk in a few genetic models in MassArray subgroup and other subgroup. Out of the 35 qualified case-control studies, only one study did not satisfy the HWE. After removing this study, the statistically significant association between rs2274223 polymorphism and cancer risk still existed [AG vs. AA: OR 1.169, 95% CI 1.083–1.262 (*P* < 0.001); GG vs. AA: OR 1.341, 95% CI 1.158–1.554 (*P* < 0.001); AG+GG vs. AA: OR 1.190, 95% CI 1.098–1.288 (*P* < 0.001); GG vs. AA+AG: OR 1.254, 95% CI 1.098–1.432 (*P* = 0.001); G vs. A: OR 1.157, 95% CI 1.083–1.236 (*P* < 0.001)].

**Table 2:** Meta-analysis of the relationship between *PLCE1* rs2274223 polymorphism and cancer risk.

**SNP**		***n***	**Association results**		**Heterogeneity**		
			**OR (95% CI)**	***P* (Z-t)**	***P* (Q-t)**	***I*^**2**^ (%)**	**Model**
**rs2274223**							
Total	AG vs. AA	35	1.168 (1.084, 1.259)	<0.001	<0.001	56.4	R
	GG vs. AA	35	1.351 (1.163, 1.570)	<0.001	<0.001	58.5	R
	AG+GG vs. AA	35	1.193 (1.103, 1.290)	<0.001	<0.001	63.8	R
	GG vs. AA+AG	35	1.262 (1.102, 1.446)	0.001	<0.001	53.3	R
	G vs. A	35	1.163 (1.089, 1.242)	<0.001	<0.001	68.3	R
**CANCER TYPE**							
GC	AG vs. AA	10	1.138 (0.979, 1.323)	0.093	<0.001	73.0	R
	GG vs. AA	10	1.317 (1.041, 1.667)	0.022	0.012	57.3	R
	AG+GG vs. AA	10	1.163 (1.002, 1.350)	0.047	<0.001	74.8	R
	GG vs. AA+AG	10	1.271 (1.114, 1.449)	<0.001	0.037	49.6	F
	G vs. A	10	1.144 (1.018, 1.286)	0.023	<0.001	74.1	R
EC	AG vs. AA	17	1.247 (1.157, 1.344)	<0.001	0.067	36.4	F
	GG vs. AA	17	1.542 (1.247, 1.907)	<0.001	0.008	51.0	R
	AG+GG vs. AA	17	1.266 (1.133, 1.415)	<0.001	0.004	54.1	R
	GG vs. AA+AG	17	1.356 (1.192, 1.544)	<0.001	0.043	40.5	F
	G vs. A	17	1.226 (1.112, 1.351)	<0.001	<0.001	63.6	R
CRC	AG vs. AA	4	1.152 (0.963, 1.379)	0.121	0.161	41.8	F
	GG vs. AA	4	1.079 (0.406, 2.868)	0.879	0.002	79.4	R
	AG+GG vs. AA	4	1.118 (0.818, 1.528)	0.483	0.024	68.2	R
	GG vs. AA+AG	4	1.007 (0.412, 2.459)	0.988	0.005	76.6	R
	G vs. A	4	1.095 (0.786, 1.525)	0.590	0.001	81.2	R
HNC	AG vs. AA	2	1.091 (0.947, 1.257)	0.225	0.544	0.0	F
	GG vs. AA	2	1.289 (0.839, 1.981)	0.246	0.136	55.0	R
	AG+GG vs. AA	2	1.111 (0.971, 1.271)	0.127	0.875	0.0	F
	GG vs. AA+AG	2	1.251 (0.773, 2.025)	0.362	0.091	65.0	R
	G vs. A	2	1.092 (0.983, 1.213)	0.100	0.580	0.0	F
**ETHNICITY**							
Asian	AG vs. AA	21	1.221 (1.102, 1.352)	<0.001	<0.001	67.8	R
	GG vs. AA	21	1.665 (1.381, 2.006)	<0.001	0.001	56.1	R
	AG+GG vs. AA	21	1.270 (1.142, 1.412)	<0.001	<0.001	72.8	R
	GG vs. AA+AG	21	1.465 (1.316, 1.632)	<0.001	0.016	44.2	F
	G vs. A	21	1.251 (1.145, 1.366)	<0.001	<0.001	74.0	R
Caucasian	AG vs. AA	12	1.078 (0.979, 1.187)	0.128	0.373	7.4	F
	GG vs. AA	12	0.991 (0.840, 1.169)	0.913	0.312	13.6	F
	AG+GG vs. AA	12	1.066 (0.972, 1.169)	0.177	0.401	4.5	F
	GG vs. AA+AG	12	0.955 (0.816, 1.117)	0.561	0.230	21.7	F
	G vs. A	12	1.027 (0.958, 1.101)	0.448	0.437	0.7	F
**SOURCE OF CONTROL**							
HB	AG vs. AA	19	1.188 (1.106, 1.277)	<0.001	0.112	29.4	F
	GG vs. AA	19	1.289 (1.009, 1.647)	0.042	<0.001	60.4	R
	AG+GG vs. AA	19	1.206 (1.126, 1.291)	<0.001	0.018	45.1	F
	GG vs. AA+AG	19	1.199 (0.951, 1.512)	0.126	0.001	59.0	R
	G vs. A	19	1.150 (1.050, 1.259)	0.003	<0.001	59.6	R
PB	AG vs. AA	15	1.169 (1.028, 1.329)	0.017	<0.001	73.0	R
	GG vs. AA	15	1.448 (1.184, 1.770)	<0.001	0.003	58.0	R
	AG+GG vs. AA	15	1.203 (1.054, 1.373)	0.006	<0.001	76.9	R
	GG vs. AA+AG	15	1.352 (1.205, 1.516)	<0.001	0.039	43.1	F
	G vs. A	15	1.184 (1.066, 1.316)	0.002	<0.001	77.1	R
**GENOTYPING METHOD**							
TaqMan	AG vs. AA	15	1.219 (1.144, 1.298)	<0.001	0.063	38.7	F
	GG vs. AA	15	1.369 (1.220, 1.537)	<0.001	0.048	41.3	F
	AG+GG vs. AA	15	1.248 (1.175, 1.325)	<0.001	0.016	49.1	F
	GG vs. AA+AG	15	1.256 (1.125, 1.401)	<0.001	0.108	32.5	F
	G vs. A	15	1.169 (1.082, 1.264)	<0.001	0.003	57.0	R
PCR	AG vs. AA	11	1.278 (1.163, 1.405)	<0.001	0.222	23.2	F
	GG vs. AA	11	1.196 (0.846, 1.692)	0.311	0.001	66.9	R
	AG+GG vs. AA	11	1.290 (1.178, 1.412)	<0.001	0.040	47.5	F
	GG vs. AA+AG	11	1.089 (0.788, 1.505)	0.606	0.002	64.4	R
	G vs. A	11	1.183 (1.041, 1.344)	0.010	0.001	66.0	R
MassArray	AG vs. AA	4	1.260 (1.065, 1.491)	0.007	0.255	26.0	F
	GG vs. AA	4	1.456 (0.626, 3.384)	0.383	0.002	79.9	R
	AG+GG vs. AA	4	1.270 (0.955, 1.688)	0.101	0.027	67.3	R
	GG vs. AA+AG	4	1.343 (0.617, 2.922)	0.458	0.005	77.0	R
	G vs. A	4	1.219 (0.902, 1.648)	0.197	0.002	80.3	R
Other	AG vs. AA	5	0.892 (0.801, 0.993)	0.036	0.105	47.7	F
	GG vs. AA	5	1.607 (0.929, 2.777)	0.090	0.034	61.5	R
	AG+GG vs. AA	5	0.979 (0.768, 1.249)	0.866	0.031	62.3	R
	GG vs. AA+AG	5	1.633 (1.006, 2.651)	0.047	0.074	53.2	R
	G vs. A	5	1.063 (0.851, 1.328)	0.591	0.010	70.1	R
**HWE**							
*P* > 0.05	AG vs. AA	34	1.169 (1.083, 1.262)	<0.001	<0.001	57.6	R
	GG vs. AA	34	1.341 (1.158, 1.554)	<0.001	<0.001	57.6	R
	AG+GG vs. AA	34	1.190 (1.098, 1.288)	<0.001	<0.001	64.8	R
	GG vs. AA+AG	34	1.254 (1.098, 1.432)	0.001	<0.001	51.8	R
	G vs. A	34	1.157 (1.083, 1.236)	<0.001	<0.001	68.6	R

*The results were calculated according to random model if I^2^ > 50%. GC, Gastric cancer; HNC, Head and neck cancer; CRC, Colorectal cancer; EC, Esophageal cancer; HB, Hospital-based; PB, Population-based; R, Random effect model; F, Fixed effect model*.

### Meta-Analysis of the Association Between *PLCE1* rs3765524 Polymorphism and Cancer Susceptibility

**Figure 3:**
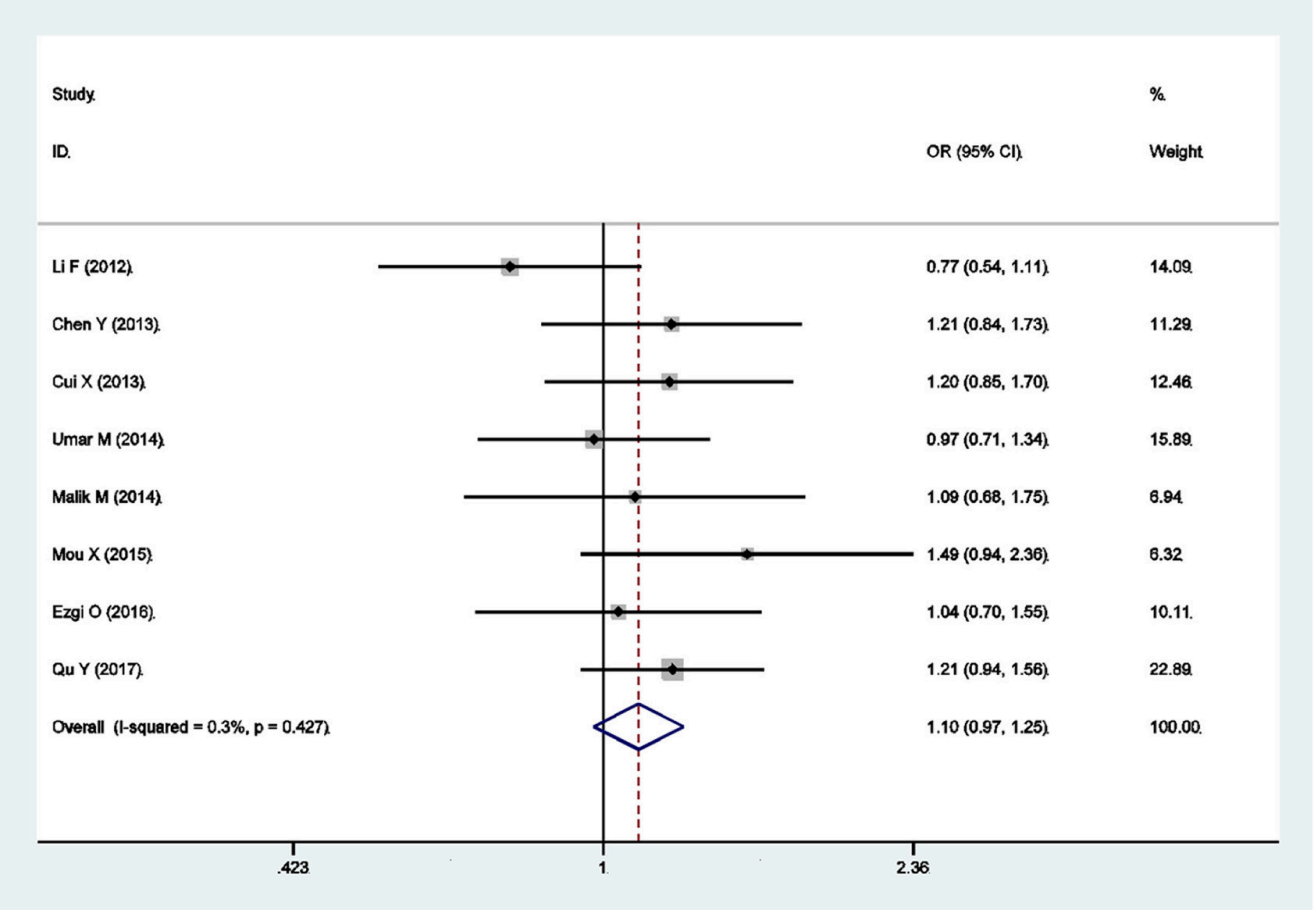
Forest plots for rs3765524 polymorphism and cancer risk in dominant model (CT+TT vs. CC).

There were eight qualified case-control studies in this meta-analysis, which assessed the relationship between the *PLCE1* rs3765524 polymorphism and cancer susceptibility. The results of meta-analysis on the relationship between the *PLCE1* rs3765524 polymorphism and cancer risk are summarized in Table 3 and Figure 3. The association between the rs3765524 polymorphism and overall cancer risk was identified in one genetic model [CT vs. CC: OR 0.681, 95% CI 0.523–0.886 (*P* = 0.004)]. The results of subgroup analyses are shown in Table 3. The results of subgroup analyses based on cancer type showed that the rs3765524 polymorphism was associated with risk of esophageal cancer in two genetic models [CT vs. CC: OR 0.611, 95% CI 0.515–0.726 (*P* < 0.001); T vs. C: OR 1.154, 95% CI 1.014–1.313 (*P* = 0.029)]. In addition, the rs3765524 polymorphism was associated with colorectal cancer susceptibility in the specific genetic models [TT vs. CC: OR 0.431, 95% CI 0.229–0.811 (*P* = 0.009); TT vs. CT+CC: OR 0.429, 95% CI 0.232–0.794 (*P* = 0.007)]. However, the observed relationship between rs3765524 polymorphism and risk of gastric cancer was not statistically significant. Subgroup analyses according to ethnicity identified an association between the rs3765524 polymorphism and cancer risk in Asians [CT vs. CC: OR 0.579, 95% CI 0.492–0.680 (*P* < 0.001)]. However, the association was not statistically significant in the Caucasian population. The results of stratified analyses based on the source of controls showed that the CT genotype of rs3765524 decreased cancer susceptibility in the population-based subgroup relative to CC genotype [CT vs. CC: OR 0.568, 95% CI 0.371–0.870 (*P* = 0.009)]. However, the results of stratified analyses were not statistically significant in the hospital-based subgroup. The results of subgroup analyses based on genotyping method indicated that the rs3765524 polymorphism is not associated with tumor risk in each subgroup. Finally, we carried out subgroup analyses based on HWE. In the subgroup whose genotype frequencies among controls was consistent with HWE, the rs3765524 polymorphism was associated with cancer risk in only one genetic model [CT vs. CC: OR 0.594, 95% CI 0.511–0.691 (*P* < 0.001)]. However, the results were not statistically significant in the subgroup whose genotype frequencies among controls were not consistent with HWE.

**Table 3:** Meta-analysis of the association between *PLCE1* rs3765524 polymorphism and cancer susceptibility.

**SNP**		***n***	**Association results**		**Heterogeneity**		
			**OR (95% CI)**	***P* (Z-t)**	***P* (Q-t)**	***I*^**2**^ (%)**	**Model**
**rs3765524**							
Total	CT vs. CC	8	0.681 (0.523, 0.886)	0.004	0.001	71.5	R
	TT vs. CC	8	1.006 (0.766, 1.322)	0.965	0.183	30.6	F
	CT+TT vs. CC	8	1.103 (0.974, 1.249)	0.121	0.427	0.3	F
	TT vs. CT+CC	8	0.949 (0.726, 1.240)	0.701	0.207	27.7	F
	T vs. C	8	1.072 (0.968, 1.186)	0.180	0.215	26.7	F
**CANCER TYPE**							
GC	CT vs. CC	2	0.677 (0.458, 0.999)	0.050	0.686	0.0	F
	TT vs. CC	2	1.127 (0.644, 1.972)	0.676	0.788	0.0	F
	CT+TT vs. CC	2	1.283 (0.923, 1.781)	0.138	0.355	0.0	F
	TT vs. CT+CC	2	0.994 (0.576, 1.716)	0.983	0.594	0.0	F
	T vs. C	2	1.176 (0.906, 1.526)	0.223	0.677	0.0	F
EC	CT vs. CC	4	0.611 (0.515, 0.726)	<0.001	0.164	41.3	F
	TT vs. CC	4	1.334 (0.920, 1.936)	0.129	0.981	0.0	F
	CT+TT vs. CC	4	1.149 (0.984, 1.342)	0.079	0.725	0.0	F
	TT vs. CT+CC	4	1.277 (0.885, 1.843)	0.191	0.995	0.0	F
	T vs. C	4	1.154 (1.014, 1.313)	0.029	0.937	0.0	F
CRC	CT vs. CC	2	0.449 (0.300, 0.672)	0.707	<0.001	93.9	R
	TT vs. CC	2	0.431 (0.229, 0.811)	0.009	0.271	17.5	F
	CT+TT vs. CC	2	0.885 (0.678, 1.156)	0.372	0.274	16.4	F
	TT vs. CT+CC	2	0.429 (0.232, 0.794)	0.007	0.337	0.0	F
	T vs. C	2	0.829 (0.672, 1.024)	0.082	0.204	38.1	F
**ETHNICITY**							
Asian	CT vs. CC	6	0.579 (0.492, 0.680)	<0.001	0.221	28.5	F
	TT vs. CC	6	1.064 (0.780, 1.451)	0.694	0.146	39.0	F
	CT+TT vs. CC	6	1.139 (0.987, 1.314)	0.075	0.289	19.1	F
	TT vs. CT+CC	6	0.998 (0.735, 1.355)	0.990	0.185	33.5	F
	T vs. C	6	1.095 (0.972, 1.233)	0.135	0.123	42.4	F
Caucasian	CT vs. CC	2	0.980 (0.469, 2.047)	0.956	0.005	87.4	R
	TT vs. CC	2	0.831 (0.468, 1.477)	0.529	0.272	17.1	F
	CT+TT vs. CC	2	1.001 (0.780, 1.285)	0.993	0.796	0.0	F
	TT vs. CT+CC	2	0.804 (0.460, 1.404)	0.442	0.204	38.0	F
	T vs. C	2	1.012 (0.834, 1.229)	0.901	0.524	0.0	F
**SOURCE OF CONTROL**							
HB	CT vs. CC	5	0.745 (0.515, 1.079)	0.119	0.002	76.6	R
	TT vs. CC	5	0.845 (0.490, 1.458)	0.545	0.088	50.6	R
	CT+TT vs. CC	5	1.000 (0.848, 1.179)	1.000	0.517	0.0	F
	TT vs. CT+CC	5	0.822 (0.573, 1.180)	0.288	0.096	49.3	F
	T vs. C	5	0.993 (0.870, 1.133)	0.914	0.168	38.0	F
PB	CT vs. CC	2	0.568 (0.371, 0.870)	0.009	0.081	67.1	R
	TT vs. CC	2	1.382 (0.829, 2.303)	0.215	0.913	0.0	F
	CT+TT vs. CC	2	1.211 (0.984, 1.490)	0.071	0.992	0.0	F
	TT vs. CT+CC	2	1.303 (0.787, 2.158)	0.303	0.929	0.0	F
	T vs. C	2	1.185 (0.994, 1.412)	0.059	1.000	0.0	F
**GENOTYPING METHOD**							
PCR	CT vs. CC	4	1.098 (0.925, 1.304)	0.285	0.778	0.0	F
	TT vs. CC	4	1.051 (0.705, 1.566)	0.808	0.488	0.0	F
	CT+TT vs. CC	4	1.099 (0.931, 1.298)	0.266	0.759	0.0	F
	TT vs. CT+CC	4	1.006 (0.680, 1.487)	0.977	0.422	0.0	F
	T vs. C	4	1.088 (0.950, 1.246)	0.225	0.678	0.0	F
MassArray	CT vs. CC	2	1.022 (0.788, 1.325)	0.869	0.275	16.1	F
	TT vs. CC	2	0.662 (0.141, 3.115)	0.601	0.012	84.0	R
	CT+TT vs. CC	2	0.968 (0.627, 1.493)	0.882	0.083	66.8	R
	TT vs. CT+CC	2	0.657 (0.155, 2.784)	0.568	0.018	82.0	R
	T vs. C	2	0.923 (0.568, 1.500)	0.747	0.020	81.6	R
Other	CT vs. CC	2	1.348 (0.994, 1.828)	0.055	0.236	28.8	F
	TT vs. CC	2	1.215 (0.729, 2.026)	0.455	0.579	0.0	F
	CT+TT vs. CC	2	1.310 (0.987, 1.740)	0.062	0.482	0.0	F
	TT vs. CT+CC	2	1.070 (0.650, 1.764)	0.789	0.431	0.0	F
	T vs. C	2	1.206 (0.961, 1.513)	0.105	0.850	0.0	F
**HWE**							
*P* > 0.05	CT vs. CC	6	0.594 (0.511, 0.691)	<0.001	0.177	34.5	F
	TT vs. CC	6	1.076 (0.782, 1.481)	0.652	0.146	38.9	F
	CT+TT vs. CC	6	1.081 (0.943, 1.239)	0.263	0.390	4.1	F
	TT vs. CT+CC	6	1.048 (0.765, 1.435)	0.772	0.197	31.8	F
	T vs. C	6	1.077 (0.962, 1.206)	0.200	0.146	38.9	F
*P* < 0.05	CT vs. CC	2	0.970 (0.424, 2.221)	0.943	0.021	81.3	R
	TT vs. CC	2	0.839 (0.496, 1.419)	0.513	0.295	9.0	F
	CT+TT vs. CC	2	1.215 (0.901, 1.639)	0.202	0.248	25.1	F
	TT vs. CT+CC	2	0.734 (0.441, 1.220)	0.232	0.382	0.0	F
	T vs. C	2	1.051 (0.839, 1.317)	0.664	0.249	24.6	F

*The results were calculated according to random model if I^2^ > 50%. GC, Gastric cancer; CRC, Colorectal cancer; EC, Esophageal cancer; HB, Hospital-based; PB, Population-based; R, Random effect model; F, Fixed effect model*.

### Meta-Analysis of the Relationship Between the *PLCE1* rs753724, rs11187842, and rs7922612 Polymorphisms and Cancer Risk

The rs753724, rs11187842, and rs7922612 polymorphisms were involved in four, four, and three case-control studies, respectively. The results of meta-analysis on the association between *PLCE1* (rs753724, rs11187842, and rs7922612) polymorphisms and cancer risk are summarized in Table 4 and Figure S1. Results indicated no significant relationship between the *PLCE1* rs753724, rs11187842, and rs7922612 polymorphisms and cancer risk. In addition, further subgroup analysis did not identify statistically significant relationships in any genetic model.

**Table 4:** Meta-analysis of the relationship between *PLCE1* rs753724, rs11187842, rs7922612 polymorphisms and cancer risk.

**SNP**		***n***	**Association results**		**Heterogeneity**		
			**OR (95% CI)**	***P* (Z-t)**	***P* (Q-t)**	***I*^**2**^ (%)**	**Model**
**rs753724**							
Total	GT vs. GG	4	1.371 (0.992, 1.893)	0.056	0.036	65.0	R
	TT vs. GG	4	1.088 (0.382, 3.098)	0.875	0.019	69.8	R
	GT+TT vs. GG	4	1.354 (0.955, 1.920)	0.089	0.013	72.2	R
	TT vs. GT+GG	4	1.008 (0.367, 2.769)	0.987	0.025	67.8	R
	T vs. G	4	1.273 (0.911, 1.780)	0.158	0.005	76.7	R
**CANCER TYPE**							
CRC	GT vs. GG	2	1.090 (0.830, 1.432)	0.536	0.232	30.0	F
	TT vs. GG	2	1.153 (0.204, 6.529)	0.872	0.010	85.0	R
	GT+TT vs. GG	2	1.104 (0.664, 1.834)	0.704	0.051	73.8	R
	TT vs. GT+GG	2	1.136 (0.217, 5.946)	0.880	0.013	83.7	R
	T vs. G	2	1.103 (0.604, 2.014)	0.750	0.008	85.8	R
**rs11187842**							
Total	CT vs. CC	4	1.177 (0.867, 1.598)	0.295	0.069	57.7	R
	TT vs. CC	4	1.066 (0.361, 3.144)	0.908	0.023	68.6	R
	CT+TT vs. CC	4	1.184 (0.894, 1.568)	0.239	0.089	54.0	R
	TT vs. CT+CC	4	1.033 (0.345, 3.093)	0.953	0.020	69.6	R
	T vs. C	4	1.157 (0.889, 1.506)	0.279	0.061	59.2	R
**CANCER TYPE**							
CRC	CT vs. CC	2	0.894 (0.669, 1.195)	0.448	0.956	0.0	F
	TT vs. CC	2	1.165 (0.199, 6.820)	0.866	0.010	84.8	R
	CT+TT vs. CC	2	0.944 (0.719, 1.240)	0.678	0.378	0.0	F
	TT vs. CT+CC	2	1.195 (0.205, 6.980)	0.843	0.010	84.9	R
	T vs. C	2	0.996 (0.651, 1.523)	0.984	0.073	68.8	R
**rs7922612**							
Total	CT vs. CC	3	0.862 (0.675, 1.100)	0.232	0.498	0.0	F
	TT vs. CC	3	0.866 (0.493, 1.520)	0.615	0.088	58.9	R
	CT+TT vs. CC	3	0.867 (0.687, 1.093)	0.228	0.244	29.0	F
	TT vs. CT+CC	3	0.863 (0.656, 1.134)	0.290	0.185	40.8	F
	T vs. C	3	0.901 (0.775, 1.049)	0.179	0.145	48.1	F
**ETHNICITY**							
Caucasian	CT vs. CC	2	0.809 (0.612, 1.070)	0.137	0.453	0.0	F
	TT vs. CC	2	0.733 (0.371, 1.448)	0.371	0.088	65.7	R
	CT+TT vs. CC	2	0.800 (0.612, 1.046)	0.103	0.227	31.4	F
	TT vs. CT+CC	2	0.807 (0.601, 1.083)	0.153	0.165	48.2	F
	T vs. C	2	0.855 (0.723, 1.011)	0.067	0.185	43.1	F

*The results were calculated according to random model if I^2^ > 50%. CRC, Colorectal cancer; HB, Hospital-based; PB, Population-base; R, Random effect model; F, Fixed effect model*.

### Heterogeneity, Sensitivity Analysis, and Publication Bias

**Figure 4:**
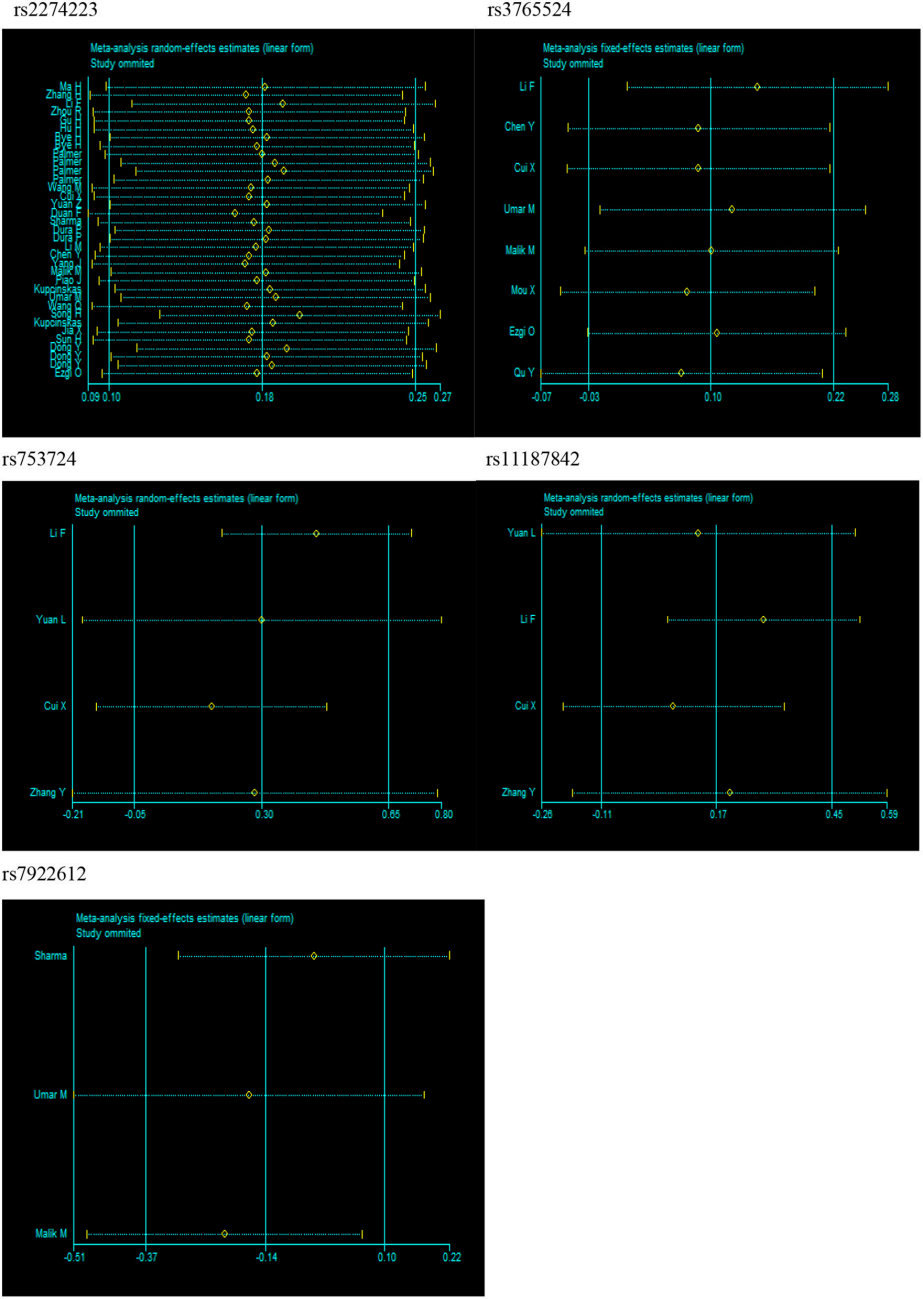
Sensitivity analysis for *PLCE1* polymorphisms and cancer risk in dominant model (rs2274223: AG+GG vs. AA; rs3765524: CT+TT vs. CC; rs753724: GT+TT vs. GG; rs11187842: CT+TT vs. CC; rs7922612: CT+TT vs. CC).

Substantial heterogeneities were identified in our meta-analysis. For example, we observed significant heterogeneity in the overall analysis for rs2274223 (*I*^2^ > 50%). Therefore, we conducted meta-regression analyses to investigate the source of heterogeneity for rs2274223. Results suggested that ethnicity is the likely source of heterogeneity for rs2274223 in the three genetic models (GG vs. AA: *P* = 0.009; GG vs. AA+AG: *P* = 0.009; G vs. A: *P* = 0.048). The genotyping method is a possible source of heterogeneity for rs2274223 in one genetic model (AG vs. AA: *P* = 0.020) (Table S1). The results of stratified analyses for rs2274223 were basically consistent with results of meta-regression. However, identifying the source of heterogeneity for rs3765524, rs753724, rs11187842, and rs7922612 was difficult based on stratified analyses. Further, results of sensitivity analysis suggested that the results of meta-analysis were not influenced by any single study in all genetic models for all five polymorphisms, which indicated that our analysis was robust and stable (Figure 4). Next, publication bias was evaluated by Egger's test and funnel plot. (Table S2 and Figure S2). The results of Egger's test showed that all *P*-values were >0.05 and that the funnel plots were relatively symmetrical, indicating no publication bias was detected in the current analysis.

## Discussion

Tumor pathogenesis involves both genetic and environmental factors. As the effects of genetic mutations on cancer continued to be revealed, many authors have focused on the associations between SNPs and cancer susceptibility. *PLCE1* is one of the members of the phospholipase C protein family, which can interact with Ras and participate in cellular signal transduction, produce secondary messengers by hydrolyzing phosphatidylinositol-4, 5-bisphosphate, and regulate cell growth, differentiation, apoptosis, and angiogenesis. (49–53) The role of *PLCE1* in cancer remains controversial. The studies of Wang et al. (54, 55) demonstrated that *PLCE1* plays a tumor suppressor role in colorectal carcinoma. However, some studies indicated that *PLCE1* acts as an oncogene in numerous cancers, such as non-small cell lung cancer (56) and head and neck cancer (57). Recent years have witnessed an increasing number of studies that investigate *PLCE1* polymorphisms and cancer susceptibility. Likewise, several meta-analyses assessed the association between *PLCE1* polymorphisms and cancer risk. However, most of these studies focused on the relationship between *PLCE1* polymorphisms and digestive tract cancer rather than the overall tumor risk.

Our current findings showed that the rs2274223 polymorphism was associated with overall tumor susceptibility in five genetic models, consistent with the results reported by Xue et al. (14). However, the current results were slightly different from those reported by Umar (58), in which the rs2274223 polymorphism showed no significant association with overall cancer susceptibility in one specific genetic model (GG vs. AG+AA). Further stratified analysis revealed that the rs2274223 polymorphism was associated with gastric cancer and esophageal cancer susceptibility, but not with other types of cancer. The above findings were consistent with those reported by Umar (58), but slightly different from the findings of Xue et al. (14), which suggested that rs2274223 polymorphism was not associated with susceptibility to gastric cancer. The results based on the esophageal cancer subgroup were consistent with the results of Wang et al. (59) and Guo et al. (60). Moreover, the results of the stratified analysis indicated that the rs2274223 polymorphism was associated with cancer susceptibility in Asians but not in Caucasians, consistent with the findings of Umar et al. (58). Results of subgroup analysis according to the source of controls identified a relationship between rs2274223 polymorphism and tumor risk regardless of whether controls were obtained from a hospital or a population and were also consistent with the findings of Umar et al. (58). For rs3765524, the results of the present meta-analysis showed that the association between the rs3765524 polymorphism and overall cancer risk was identified in only one genetic model (CT vs. CC). The results of stratified analysis indicated that the rs3765524 polymorphism was associated with colorectal cancer and esophageal cancer susceptibility but not with the other types of cancer. The above findings were distinct from those of Mocellin et al. (61), which identified an association between the rs3765524 polymorphism and gastric cancer susceptibility. Finally, our results of both the total cancer analysis or subgroup analysis indicated that the rs753724, rs11187842, and rs7922612 polymorphisms were not related to tumor risk. The consistencies between the current and previous meta-analyses might be because some of the literatures included in meta-analyses were the same. Meanwhile, the inconsistencies between the current and previous meta-analyses could be attributed to differences in inclusion criteria. For example, the present meta-analysis specifically required that the qualified studies were case-control studies, which was different from the meta-analysis of Mocellin et al. (61).

Some limitations still existed in the present analysis, though the analysis was performed carefully. First, relatively few qualified studies were included for investigating rs753724, rs11187842, and rs7922612, and some subgroups included in the stratified analysis had low sample sizes, which might have affected statistical results. Second, unified adjustment about confounders could not be carried out in our analysis because the original data were not obtained. Third, ICD-O codes of cancers from qualified studies were not obtained, and differences in cancers included in the studies might lead to biases. Finally, unpublished materials were not obtained, which might have caused publication bias, although publication bias was not detected based on Begg's funnel plots and Egger's test in this meta-analysis.

## Conclusions

Our findings indicated that the *PLCE1* rs2274223 polymorphism is significantly associated with cancer susceptibility in the overall population. On the other hand, the *PLCE1* rs753724, rs11187842, and rs7922612 polymorphisms showed no significant associations with cancer risk. In addition, the results suggested that the *PLCE1* rs3765524 polymorphism is associated with overall cancer risk under the heterozygote model (CT vs. CC).

## Author Contributions

XiaL and MJ collected the data. XiaL analyzed the data and wrote the paper. WT, XueL, and BZ read and revised the paper. All the authors supported the submission of this manuscript.

### Conflict of Interest Statement

The authors declare that the research was conducted in the absence of any commercial or financial relationships that could be construed as a potential conflict of interest.
