# Supplementary material for: Single Nucleotide Polymorphisms in PLCE1 for Cancer Risk of Different Types: A Meta-Analysis
Source: Front Oncol. 2018 Dec 11;8:613. doi: 10.3389/fonc.2018.00613 (PMC6297376; doi:10.3389/fonc.2018.00613)
Supplement: Supplementary file 1 [file Data_Sheet_1.docx]

**Table S1** The results of the meta-regression analyses for rs2274223

| Factors | AG vs. AA (*P*) | GG vs. AA (*P*) | AG+GG vs. AA (*P*) | GG vs. AA+AG (*P*) | G vs. A (*P*) |
| --- | --- | --- | --- | --- | --- |
| Ethnicity | 0.362 | 0.009 | 0.161 | 0.009 | 0.048 |
| Cancer type | 0.204 | 0.395 | 0.330 | 0.267 | 0.526 |
| Source of control | 0.942 | 0.723 | 0.902 | 0.619 | 0.811 |
| Genotyping method | 0.020 | 0.785 | 0.249 | 0.569 | 0.778 |
| HWE | 0.290 | 0.076 | 0.709 | 0.074 | 0.290 |

**Table 2** Egger’s test evaluating the publication bias

| SNPs | *P* values | 95% CI |
| --- | --- | --- |
| rs2274223 |  |  |
| AG vs. AA | 0.610 | -1.692~1.007 |
| GG vs. AA | 0.671 | -1.007~1.545 |
| AG+GG vs. AA | 0.626 | -1.838~1.122 |
| GG vs. AA+AG | 0.521 | -0.806~1.561 |
| G vs. A | 0.821 | -1.787~1.427 |
| rs3765524 |  |  |
| CT vs. CC | 0.523 | -6.170~10.906 |
| TT vs.CC | 0.073 | -12.625~0.767 |
| CT+TT vs. CC | 0.993 | -4.923~4.886 |
| TT vs. CT+CC | 0.143 | -12.152~2.239 |
| T vs. C | 0.650 | -7.944~5.348 |
| rs753724 |  |  |
| GT vs. GG | 0.193 | -10.392~27.335 |
| TT vs. GG | 0.627 | -22.281~17.080 |
| GT+TT vs. GG | 0.474 | -161.102~106.698 |
| TT vs. GT+GG | 0.557 | -21.323~15.359 |
| T vs. G | 0.382 | -145.031~85.505 |
| rs11187842 |  |  |
| CT vs. CC | 0.181 | -91.902~33.201 |
| TT vs. CC | 0.793 | -20.014~17.408 |
| CT+TT vs. CC | 0.712 | -234.243~192.142 |
| TT vs. CT+CC | 0.736 | -20.372~17.008 |
| T vs. C | 0.494 | -162.254~239.686 |
| rs7922612 |  |  |
| CT vs. CC | 0.937 | -42.434~41.781 |
| TT vs. CC | 0.453 | -68.867~82.719 |
| CT+TT vs. CC | 0.842 | -60.044~57.689 |
| TT vs. CT+CC | 0.128 | -5.535~12.507 |
| T vs. C | 0.434 | -48.298~58.654 |

rs753724


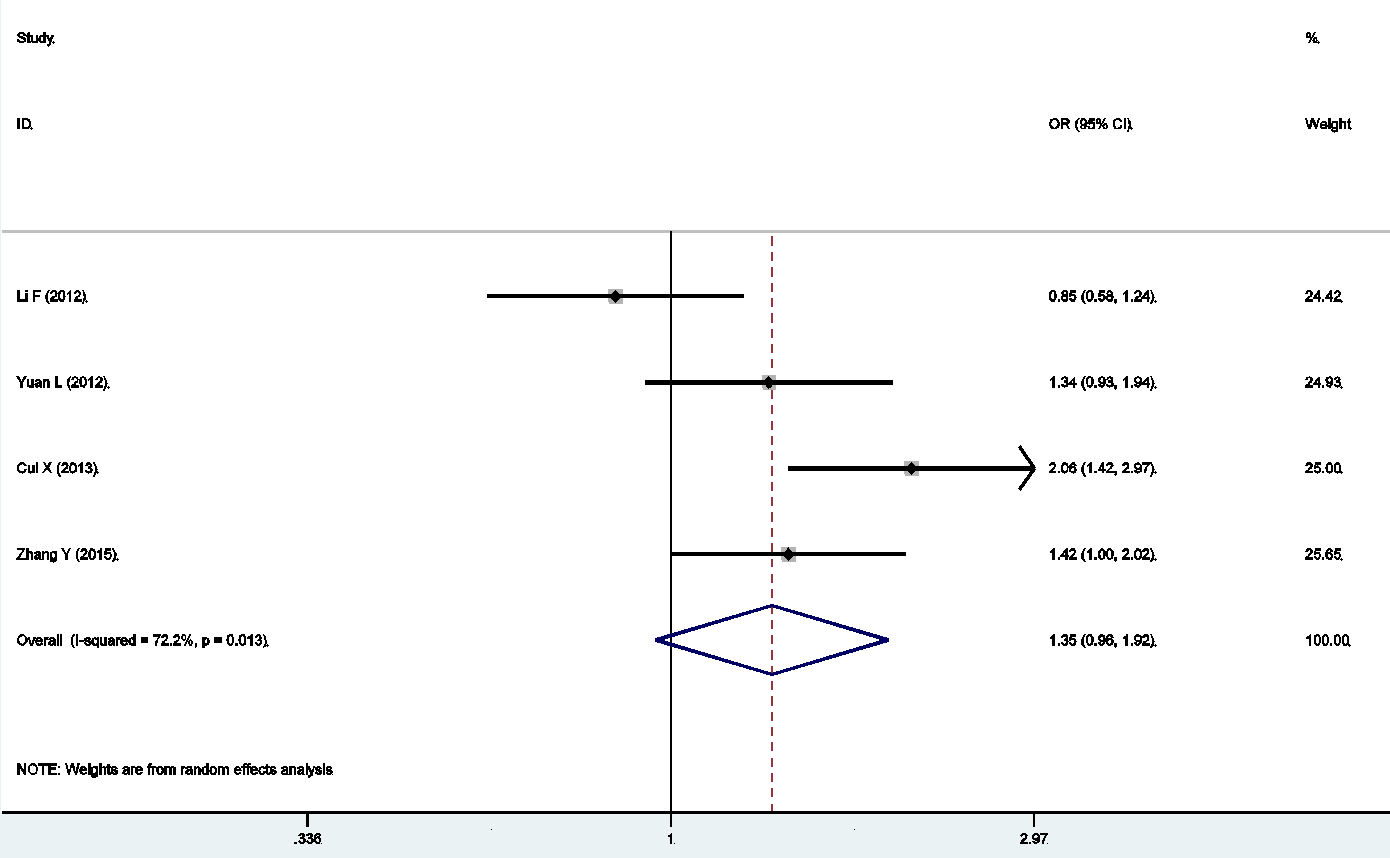


rs11187842


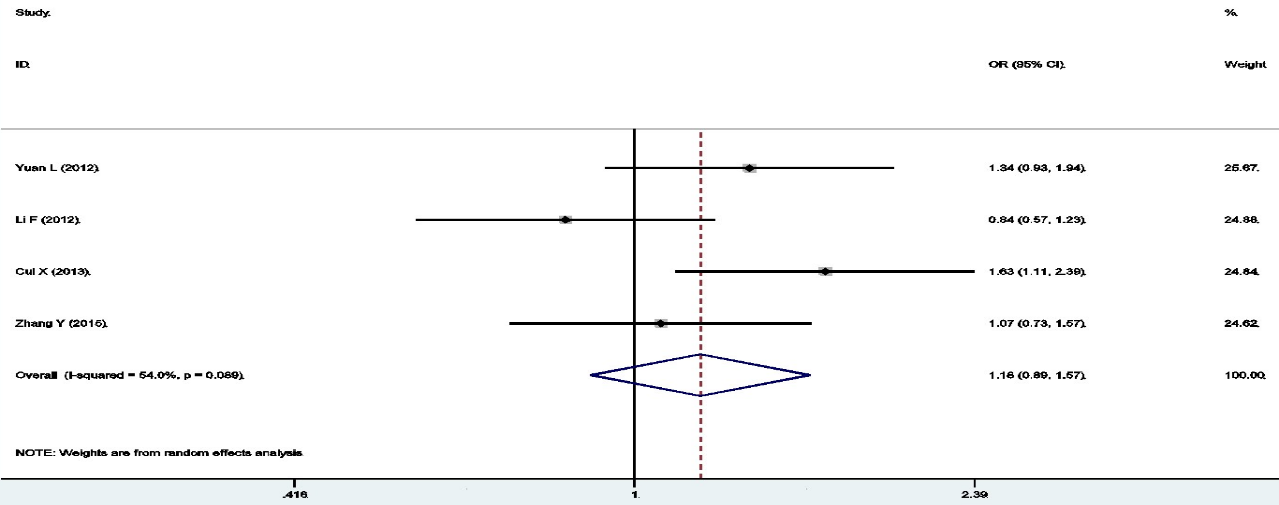


rs7922612


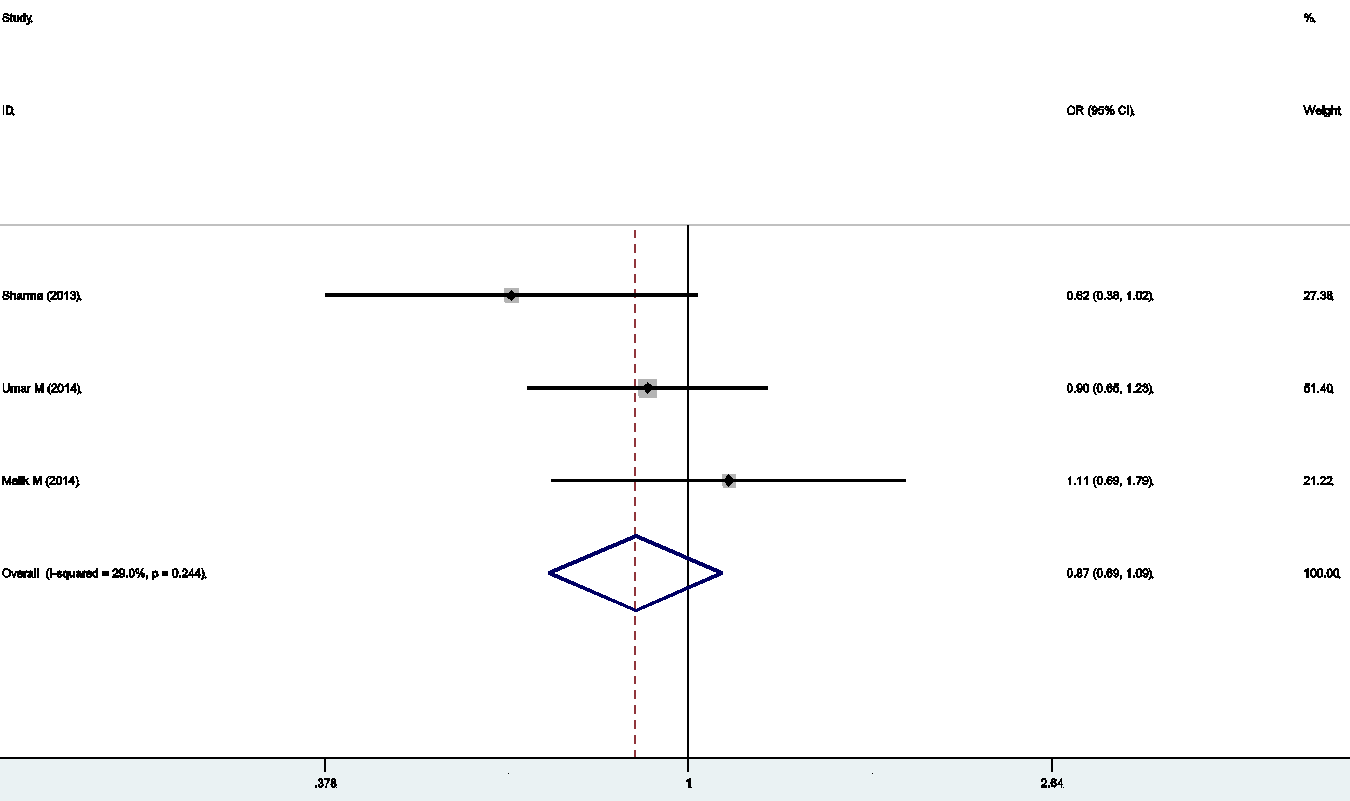


**Fig. S1** Forest plots for *PLCE1* polymorphisms and cancer risk in dominant model.

rs2274223 rs3765524


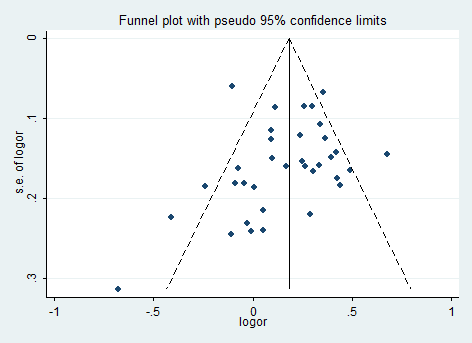

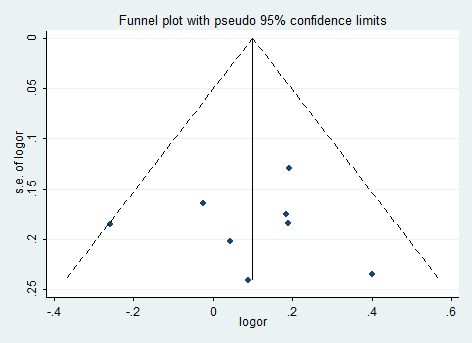


rs753724 rs11187842


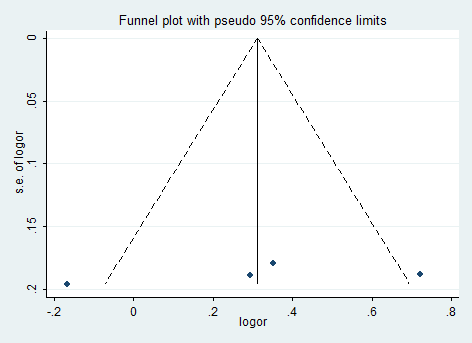

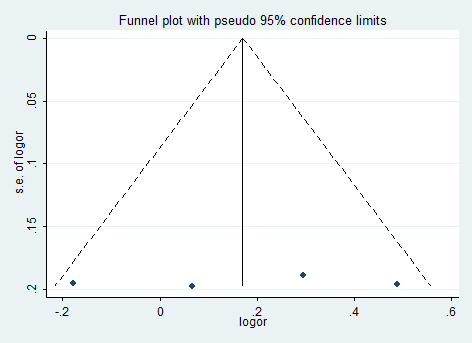


rs7922612


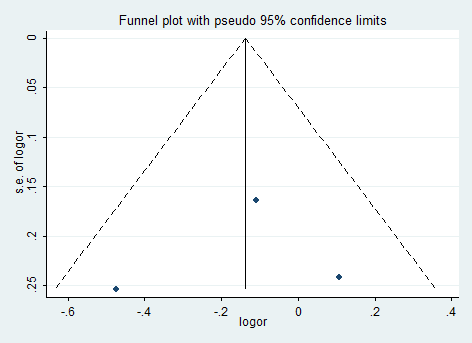


**Fig. S2** Funnel plots for *PLCE1* polymorphisms and cancer risk in the dominant model (rs2274223: AG+GG vs. AA; rs3765524: CT+TT vs. CC; rs753724: GT+TT vs. GG; rs11187842: CT+TT vs. CC; rs7922612: CT+TT vs. CC)
